# Short-term skin problems in infants aged 0–3 months affect food allergies or atopic dermatitis until 2 years of age, among infants of the general population

**DOI:** 10.1186/s13223-019-0385-7

**Published:** 2019-11-26

**Authors:** Kaori Yonezawa, Megumi Haruna

**Affiliations:** 1 Division of Health Sciences and Nursing, Department of Midwifery and Women’s Health, Graduate School of Medicine, The University of Tokyo, 7-3-1, Hongo, Bunkyo-ku, Tokyo, 113-0033 Japan; 2 Division of Nursing Systems, Department of Health Quality and Outcome Research, Global Nursing Research Center, Graduate School of Medicine, The University of Tokyo, Tokyo, Japan

**Keywords:** Dermatitis, atopic, Diaper rash, Food hypersensitivity, Infant, Skin care

## Abstract

**Background:**

This study examined whether infants aged 0–3 months exhibited long-term effects of using a moisturizer skincare intervention and whether a short-term skin problem resulted in the subsequent development of food allergies or atopic dermatitis (AD) until the age of 2 years.

**Methods:**

This study was a follow-up of a completed randomized control trial (RCT) of moisturizer skincare for infants aged 0–3 months. A self-reported questionnaire was mailed to the parents of children aged 1–2 years who had participated in the RCT. Data were analyzed using a Chi square test, by intention to treat analysis, and by multiple logistic regression.

**Results:**

Of 155 infants, 22 (14.2%) and 28 (18.1%) had food allergies and AD/eczema until 2 years of age, respectively. No significant difference was seen in food allergies or AD between the group that received moisturizer skincare intervention and the control group. On the contrary, food allergies until 2 years of age were significantly associated with short-term (4–7 days) and long-term (more than 7 days) body skin problems occurring in the first 3 months of life, a family history of AD, and the time of starting complementary food. High value of face transepidermal water loss at 3 months of age was also associated with food allergies. Moreover, a short duration of severe diaper dermatitis during the first 3 months, a family history of AD, and being male were significantly associated with AD/eczema until the age of 2 years.

**Conclusions:**

After adjusting for family history of AD, a short-term skin problem in the first 3 months of life was significantly associated with the development of food allergies or AD/eczema until the age of 2 years. Prevention or prompt treatment of skin problems in newborns is essential for preventing future allergic diseases.

*Trial registration* This was a follow-up study conducted 2 years after the completed RCT of a moisturizer skincare intervention for early infants, which was registered in the University Hospital Medical Information Network Clinical Trials Registry (UMIN000013260)

## Background

Skin conditions in early infants have received attention as a mechanism involved in the onset of infant allergies. This mechanism is described by the “dual allergen exposure hypothesis” [1], which proposes that skin barrier dysfunction causes percutaneous sensitization of allergens before an infant commences complementary food. A number of previous studies have shown that skin barrier dysfunction, particularly early onset atopic dermatitis (AD) and high transepidermal water loss (TEWL), can cause food allergies in children [2, 3]. The occurrence of eczema in the first 2 or 3 months of life is associated with food allergies at 1 and 3 years of age [3, 4]. In addition, AD severity is known to be related to subsequent food allergies [5]. Application of moisturizer to protect skin barrier function has been shown to reduce the onset of AD and sensitization to egg white among high-risk infants with a family history of AD [6, 7]. Therefore, identifying and treating skin conditions, such as AD and other skin problems, in early infanthood is important for preventing childhood allergic diseases.

However, whether the duration of skin problems in infants plays an important role in percutaneous sensitization to allergens is unclear. Skin problems can develop in various parts of the body and can appear as infantile skin problems on the face or as diaper dermatitis. However, the complete prevention of infantile skin problems may be virtually impossible. A previous study investigated the presence or absence of eczema in a patient’s past 1 month to 1 year history [3], but did not evaluate the severity or duration of skin problems and the findings were potentially subject to recall bias. However, whether differences in the duration of infant skin problems affect subsequent allergic diseases is unclear.

Therefore, to better understand the possibility of developing allergies, understanding the role of the parts of the body affected by and the duration of skin problems early in a child’s life is important. If a relationship between a short-term skin problem and subsequent allergic disease exists, there is a need for prompt medical intervention at the time an infant develops the skin problem. The relationship, therefore, between the type or duration of skin problems and subsequent allergic diseases needs to be clarified.

Many previous studies based on the dual allergen exposure hypothesis have been conducted among high-risk populations with a family history of AD. Conversely, few studies have analyzed the general population, or those considered to have a low risk of allergic disease. To understand the relationship between infantile skin problems and subsequent allergic diseases and to investigate methods for the prevention of allergic diseases, low-risk populations should also be studied.

We previously conducted a randomized control trial (RCT) of a skincare moisturizer intervention among infants aged 0–3 months that aimed to improve the skin barrier function of infants and reduce the incidence of skin problems for up to 3 months after birth [8]. Furthermore, data collected regarding the affected body parts and duration of skin problems in early infancy can be used to better understand the occurrence of skin problems in infants 0–3 months and their association with allergic diseases at the age of 2 years.

## Methods

### The aim

This study first aimed to investigate whether moisturizing skin care for infants could prevent subsequent allergic disease among the infants regardless of their family histories of AD. Second, we examined the relationship between various aspects of infantile skin problems in the first 3 months of life and the development of allergic diseases (food allergies or AD) until 2 years of age.

### Study design and setting

This was a follow-up study conducted 2 years after the completed RCT of a moisturizer skincare intervention for early infants, which was registered in the University Hospital Medical Information Network Clinical Trials Registry (UMIN000013260). The parents of these infants were recruited between March 2014 and February 2015 (when their newborns were 1–3 days old), at an obstetrics hospital in Tokyo. Skin barrier function, skin problems, and demographic data were collected during the participants’ first 3 months of life and details of that study are published elsewhere [8]. The present study was conducted by mailing self-reported follow-up questionnaires to parents whose infants were aged 1–2 years old, between March 2015 and February 2017. The questionnaire asked about allergic diseases outcomes and focused on potentially related factors.

### Participants

The inclusion criteria for the RCT were as follows: (i) newborns born at the institution at a minimum gestational age of 35 weeks, (ii) newborns born to Asian parents, (iii) newborns who received no medical treatment in the pediatric ward, and (iv) newborns who had a mother who was able to speak Japanese.

The intervention was performed between birth and 3 months of age. The intervention group performed moisturizing skincare as follows: (i) routine bathing every 2 days (reduced bathing frequency) and (ii) use of a moisturizer one or more times per day. The control group performed the common skincare regimen used in Japan, which was as follows: (i) routine bathing daily and (ii) no moisturizer. At the research hospital, midwives traditionally recommend to all mothers that they bathe their newborn daily.

### Outcome (allergic diseases until 2 years of age)

We used self-reported questionnaires administered to the parents when their infants were aged 1–2 years. The questionnaire included items concerning the child’s current skin condition (good condition, almost good condition, occasional skin problems, dry skin, have skin problems frequently and not using steroids, have skin problems frequently and using steroids, and AD diagnosis) and allergic diseases (food allergy, asthma, and other allergies). In this study, “many skin problems and using steroids” and “diagnosed atopic dermatitis” were both considered AD/eczema. We have used the following definitions of skin conditions throughout this study: (i) a “skin problem” occurred in infants aged 0–3 months and was reported by parents, (ii) “eczema” occurred in children from 3 months to 2 years old and the parents had been instructed by a physician to use topical steroid ointment, and (iii) “AD” had been diagnosed by a physician when the child was from 3 months to 2 years old. In addition, we assessed whether participants had self-reported food allergies by asking, “Does your infant have any food allergies?” If parents answered “yes”, infants were considered as having a “food allergy”. If infants had a food allergy, we asked what food allergy their infants had, and when the parents recognized their infants had a food allergy.

### Skin barrier function

The infant’s skin barrier function was evaluated in the previous RCT by measuring the TEWL values (Tewameter TM300; Courage + Khazaka Electronic, Cologne, Germany) when the infants were 4 days old (baseline), 1 month old, and 3 months old (main outcome of the RCT). These values were calculated by taking the average of the TEWL value of the forehead and cheek. All measurements were conducted in hospital rooms with the temperature controlled at 24–28 °C for at least 5 min after the infant entered the room and at least 2 h after the parents had cared for their infant’s skin.

### Skin problems (infants aged 0–3 months)

We evaluated skin problems based on the infants’ skin diaries, which were completed by their parents. The parents recorded skin conditions assessed as follows: redness (none, small area, or middle to large area); papules (none or only 1–2, small area, or middle to large area); dryness (none, small area, or middle area to cracks); and desquamation (none, small area, or large area). The condition was recorded for 3 areas of their infant: the diaper area, face, and body (defined as an area other than face or diaper area, including limbs, chest, abdomen, and back).

The presence of skin problems on the face or body was determined using an original scale based on the Neonatal Skin Condition Score [9], which rates a skin condition between 3 and 9 points. In our study, moderate and severe skin problems were defined as at least 5 (level 3 on the scale) and 7 (level 5 on the scale) points, respectively. Thus, in this study, we defined a “severe body skin problem” as extensive (over 50% of the body) dryness on the face or on the body (arms or trunk). Parents were meant to choose three patterns: (1) moderate area of cracked dry skin and papules (moderate to large area); (2) moderate area of cracked dry skin, small area of papules, and small area of desquamation; and (3) small area of dry skin and moderate to large area of papules and small area of desquamation.

The presence of diaper dermatitis was determined using the diaper rash and erythema scoring scale [10], which rates diaper dermatitis on 7 levels ranging from none to severe. Diaper dermatitis was defined as moderate (level 5 on the erythema scoring scale) when the following were observed: redness over a large area (10–50%) or very intense redness over a very small area (< 2%), and/or single to several areas of papules (10–50%) with 0–5 pustules, and slight desquamation or edema [10]. For “severe diaper dermatitis” the diaper area must have had a moderate to large area of redness, or a small or moderate to large area of papules, and a small area or large area of desquamation.

In the present study, we categorized the duration of skin problems as follows: almost no skin problem (0–3 days), short-term skin problem (4–7 days), and long-term skin problem (more than 7 days). We hypothesized that skin problems lasting several days (more than 3 days) in a row meant that the infants had experienced repeated cutaneous exposure that caused an allergy. In addition, not only long durations of skin problems, but also short-term skin problems within 1 week were high risks for experiencing cutaneous exposure.

### Demographic data and external irritant exposure

The following demographic data were collected from the newborns’ medical charts: delivery method, mother’s parity, sex, gestational age, and birth weight. The family history of AD was collected from questionnaires completed during the previous RCT. The 1 year of age questionnaire included questions regarding when the infant began to eat complementary food.

### Statistical methods

First, we compared the onset of allergic diseases between infants with and without a family history of AD using the Chi square test. Next, univariate analyses for food allergies or AD/eczema until 2 years of age and the characteristics of the infants’ skin problems were performed using the Chi square test or Student’s t-test.

Possible long-term effects of the previously-described RCT intervention on the development of subsequent outcomes (food allergies or AD/eczema) were analyzed using the Chi square test in intention to treat and per protocol analyses (applying emollient more than 0.7 times per day from 0 to 3 months of age).

Lastly, the relationship between skin problems from 0 to 3 months of age and food allergies or AD/eczema until 2 years old was assessed using multiple logistic regression analysis. The analysis was adjusted for the effects of variables found to be associated (*p* < 0.1) with the presence of diaper dermatitis in the univariate analysis.

The statistical analyses were performed using the SPSS statistical software version 22.0 (IBM Corp., Armonk, NY, USA). All *p*-values were two sided, and a *p*-value < 0.05 was considered statistically significant.

## Results

### Participants

We obtained consent from 227 parents to participate in the present study, and 200 completed a 3-month diary of their infants’ skin conditions. Of these 200 parents, 15 were excluded because they had moved to new (unknown) addresses before the infant was 2 years of age, and the follow-up questionnaire could not be mailed. Therefore, we sent the questionnaire to 185 parents, of whom 156 (84.3%) returned and completed both questionnaires, for 1 year and 2 years of age. One participant was excluded from analysis because she failed to answer the question of whether she had a family history of AD. Therefore, data from a final total of 155 infants were analyzed.

### Allergic disease outcomes

Of the 155 infants, 22 infants (14.2%) had a food allergy until 2 years of age. The most common allergen was egg with 19 infants (86.4% of infants with a food allergy), followed by cow milk with 9 infants (40.9% of infants with a food allergy). Eight infants (36.3% of infants with a food allergy) had multiple food allergies (details of the allergens are shown in Additional file 1: Table S1).

A total of 15 infants were diagnosed with AD by a physician until 2 years of age. In addition, 13 infants were not diagnosed with AD by a physician, but their parents reported that the infants often had eczema and that they were instructed to use steroid ointment by a physician. Therefore, we considered 28 infants (18.1%) to have had AD/eczema. Eleven infants (7.1%) had both AD/eczema and a food allergy.

Table 1 summarizes the relationship between the allergy outcome data and the family history of AD. Our findings confirmed that a family history of AD was strongly associated with the AD/eczema and food allergy outcomes in the infants. In contrast, we detected no meaningful relationship between asthma and a family history of AD.

**Table 1:** Relationship between family history of AD and allergy diseases

	Family history of AD		*p*-value^†^
	Yes (n = 42)	No (n = 113)	
AD (diagnosed until 2 years old)	11 (26.2%)	4 (3.5%)	< 0.001
AD/eczema (until 2 years old)^‡^	15 (35.7%)	13 (11.5%)	< 0.001
Food allergy (at 2 years old)	10 (23.8%)	5 (4.4%)	< 0.001
Food allergy (until 2 years old)	12 (28.6%)	10 (8.8%)	0.002
Asthma	2 (4.8%)	6 (5.3%)	0.891
Other allergy	4 (9.5%)	3 (2.7%)	0.067

Data are presented as n (%)

^†^ Calculated using a Chi square test

^‡^Defined as “eczema” when parents were instructed to use steroid ointment by a physician to treat their infants’ skin condition

### Relationship between allergic diseases until 2 years old and the moisturizer intervention in the first 3 months of life

No significant differences were found in the development of a food allergy and AD/eczema until 2 years of age between the moisturizer RCT intervention and control groups in the intention to treat analysis (Table 2). Similarly, no significant differences were found in the per protocol analysis, which indicated that the development of food allergies and AD/eczema until 2 years of age did not differ between the moisturizer group and the control group (Additional file 1: Table S2).

**Table 2:** Relationship between food allergy or AD/eczema until 2 years old and moisturizer intervention in the first 3 months of life

	Total	Intervention group^†^	Control group^‡^	*p*-value^§^
	n = 155	n = 67	n = 88	
Food allergy	22 (14.2)	11 (16.4)	11 (12.5)	0.489
AD/eczema	28 (18.1)	14 (20.9)	14 (15.9)	0.424

Data are presented as n (%)

^† ^The intervention group performed moisturizing skincare

^‡ ^The control group performed the common skincare regimen

^§^ Calculated using a Chi square test

### Relationship between allergic diseases until 2 years of age and duration of skin problems in the first 3 months of life

Table 3 shows the characteristics of the participants and their allergic diseases until 2 years of age. Table 4 shows the relationship between the duration of skin problems from 0 to 3 months of age and allergic diseases. The duration of the infants’ severe body skin problems was significantly related to a food allergy until 2 years of age (*p *= 0.001).

**Table 3:** Relationship between characteristics of the participants and food allergy or AD/eczema

At birth	Food allergy		*p*-value	AD/eczema		*p*-value
	Yes (n = 22)	No (n = 133)		Yes (n = 28)	No (n = 127)	
Vaginal delivery	20 (15.4)	110 (84.6)	0.333^§^	23 (17.7)	107 (82.3)	0.784^§^
Parity: primipara	14 (14.4)	83 (85.6)	0.912^§^	16 (16.5)	81 (83.5)	0.511^§^
Family history of AD^†^	12 (28.6)	30 (71.4)	0.002^§^	15 (35.7)	27 (64.3)	*<* 0.001^§^
Both parents had AD	6 (85.7)	1 (14.3)	*<* *0.001*^§^	4 (57.1)	3 (42.9)	0.003^§^
Sex: male	15 (17.9)	69 (82.1)	0.155^§^	22 (26.2)	62 (73.8)	0.004^§^
Gestational age (days)	278.6 ± 8.2	275.6 ± 8.7	0.131^||^	276.3 ± 9.3	275.9 ± 8.6	0.826^||^
Birth weight (g)	3090 ± 443	2963 ± 326	0.110^||^	3079 ± 410	2959 ± 328	0.096^||^
TEWL (g/m^2^/h): Face at 4 days of age^‡^	7.86 ± 2.68	7.03 ± 2.28	0.125^||^	6.87 ± 2.00	7.21 ± 2.42	0.489^||^
Complementary food started after 6 months of age (n = 153)	17 (19.5)	70 (80.5)	0.037^§^	17 (19.5)	70 (80.5)	0.649^§^
TEWL (g/m^2^/h) at 1 month of age: Face^‡^	16.76 ± 7.16	11.87 ± 7.43	0.005^||^	15.03 ± 9.20	12.03 ± 7.08	0.056^||^
TEWL (g/m^2^/h) at 3 months of age: Face^‡^	23.48 ± 10.91	14.78 ± 7.21	0.001^||^	19.79 ± 10.47	15.19 ± 7.64	0.008^||^

Data are presented as n (%) or mean ± standard deviation

^† ^A family history of atopic dermatitis including the mother, father, and siblings

^‡^ TEWL, transdermal water loss; calculated as an average of the forehead and cheek

^§^ Calculated using a Chi square test

^||^ Calculated using a Student's t-test

**Table 4:** Duration of skin problems in the first 3 months of life

Duration of skin problems	Food allergy (n = 22; 14.2%)			*p*-value^†^	AD/eczema (n = 28; 18.1%)			*p*-value^†^
	Nothing (0–3 days)	Short-term (4–7 days)	Long-term (more than 7 days)		Nothing (0–3 days)	Short-term (4–7 days)	Long-term (more than 7 days)	
Had severe diaper dermatitis^‡^	18 (12.9)	2 (22.2)	2 (33.3)	0.288	22 (15.7)	4 (44.4)	2 (33.3)	0.058
Had moderate face skin problems^§^	4 (8.2)	0 (0.0)	18 (18.6)	0.107	7 (14.3)	0 (0.0)	21 (21.6)	0.192
Had severe face skin problems^§^	17 (13.0)	1 (14.3)	4 (23.5)	0.503	21 (16.0)	3 (42.9)	4 (23.5)	0.164
Had moderate body skin problems^§^	9 (11.0)	3 (17.6)	10 (17.9)	0.477	13 (15.9)	1 (5.9)	14 (25.0)	0.150
Had severe body skin problems^§^	17 (11.6)	2 (66.7)	3 (50.0)	0.001	25 (17.1)	1 (33.3)	2 (33.3)	0.471

Data are presented as n (%), where (%) indicates the incidence rate of food allergy or AD/eczema

^†^ Calculated using a Chi square test

^‡^Dermatitis was assessed using the diaper rash and erythema scoring scale. In this study, at least a moderate score (2.0) determined severe diaper dermatitis

^§^Skin problems were assessed using the Neonatal Skin Condition Score. In this study, moderate and severe skin problems were defined as at least 5 (level 3 on the scale) and 7 (level 5 on the scale) points, respectively

Finally, 4 factors were found to be significantly related to food allergies by multiple logistic regression: a family history of AD (adjusted OR [aOR], 4.59; 95% CI 1.66–12.72); a short-term severe body skin problem (4–7 days) from 0 to 3 months of age (aOR, 27.55; 95% CI 1.92–395.47); a long-term severe body skin problem (more than 7 days) from 0 to 3 months of age (aOR, 8.36; 95% CI 1.20–58.23), and starting complementary food after 6 months of age (aOR, 3.79; 95% CI 1.16–12.46) (Table 5). These factors were significantly associated after adjusting for the moisturizer intervention group in the RCT.

**Table 5:** Risk factors significantly associated with food allergy (n = 153)

	COR	95% CI	*p*-value	AOR	95% CI	*p*-value
Family history of AD	4.12	1.62–10.47	0.003	4.59	1.66–12.72	0.003
Had short-term severe body skin problems (4–7 days) in the first 3 months of life^†^	13.20	1.14–152.37	0.039	27.55	1.92–395.47	0.015
Had long-term severe body skin problems (more than 7 days) in the first 3 months of life^†^	6.84	1.29–36.39	0.024	8.36	1.20–58.23	0.032
Complementary food started after 6 months of age	2.96	1.03–8.51	0.044	3.79	1.16–12.46	0.028

*COR* crude odds ratio, *95% CI* 95% confidence interval, *AOR* adjusted odds ratio (adjusted for 4 variables in this table)

^†^Assessed using logistic regression analysis (food allergy, n = 22; no food allergy, n = 131); n = 2 were missing information about when the infant began to eat complementary food

Moreover, high values of TEWL at 1 or 3 months of age, the main outcomes of the moisturizer intervention trial, were significantly related to AD/eczema after adjusting for a family history of AD (Additional file 1: Tables S3, S4). However, the face TEWL at 3 months of age was excluded from the multiple logistic regression because of the significant relationship (as determined by the t-test) between the factors “had severe body skin problems for more than 3 days from 0 to 3 months of age” and the TEWL at 3 months of age (had skin problem vs none; 22.52 g/m^2^/h vs 14.83 g/m^2^/h, *p* < 0.001).

Furthermore, 3 factors were found to be significantly associated with AD/eczema until 2 years of age in the multiple logistic regression analysis: a family history of AD (aOR, 5.67; 95% CI 2.18–14.71), diaper dermatitis lasting 4–7 days between 0 and 3 months of age (aOR, 7.84; 95% CI 1.58–39.04), and male sex (aOR, 4.30; 95% CI 1.53–12.03) (Table 6). These factors were significantly associated after adjusting for the moisturizer intervention group in the RCT.

**Table 6:** Risk factors significantly associated with AD/eczema (n = 155)

	COR	95% CI	*p*-value	AOR	95% CI	*p*-value
Family history of AD	4.27	1.82–10.06	0.001	5.67	2.18–14.71	< 0.001
Had short-term diaper dermatitis in the first 3 months of life (4–7 days)^†^	4.07	1.02–16.26	0.047	7.84	1.58–39.04	0.012
Had long-term diaper dermatitis in the first 3 months of life (more than 7 days)^†^	2.37	0.41–13.60	0.335	2.20	0.28–17.17	0.452
Sex: male	3.84	1.46–10.11	0.006	4.30	1.53–12.03	0.002

*COR* crude odds ratio, *95% CI* 95% confidence interval, *AOR* adjusted odds ratio (adjusted for 4 variables in this table)

^†^Assessed using logistic regression analysis (AD/eczema n = 28, non-AD/eczema n = 127)

## Discussion

We found a significant relationship between short-term skin problems in infants from 0 to 3 months of age and food allergies or AD/eczema until 2 years of age after adjusting for a family history of AD. However, no significant differences in the development of food allergies and AD/eczema until 2 years of age were noted between infants who received the moisturizer intervention from 0 to 3 months of age and the control group.

A previous study showed that having a skin problem lasting more than 2 weeks was related to a subsequent egg allergy [6]. A novel finding of the present study was that skin problems lasting as short as 4–7 days in infants aged 0–3 months increased the risk of being exposed to allergens by percutaneous sensitization. In addition, moderate skin problems did not relate to subsequent food allergies. This might mean that the treatment of moderate skin problems before they become worse (severe skin problems) could prevent percutaneous sensitization. This finding highlights the importance of not only preventing but also treating skin problems promptly in infants to prevent the development of food allergies.

Although our findings showed that diaper dermatitis from 0 to 3 months of age was related to AD until 2 years of age, causality was difficult to determine. Although factors other than short-term severe diaper dermatitis might predict AD development, exposure of skin in the diaper area to allergens by percutaneous sensitization would be unlikely [1]. Because skin in the diaper area is often subjected to irritation [11], the skin here is vulnerable to skin barrier dysfunction, and thus, diaper dermatitis may be more likely to lead to the future onset of AD than other skin problems in infancy. Therefore, diaper dermatitis may indicate a congenital skin vulnerability that may be related to the development of AD.

In this study, the long-term effect of skincare from 0 to 3 months of age on the subsequent development of allergic diseases was not clear. In the previous study, moisturizer was shown to prevent AD among infants with a family history of AD [6]. However, in this study among infants in the general population, we found no such relationship. We speculate the reason for this may be, first, the small sample size in our study [8]. Secondly, although the existence of infantile skin problems has been shown to be important in the development of allergic diseases [4], it is possible that skin moisturizer may not be necessary for infants who do not have a family history of AD. Thus, a high risk approach might be appropriate in preventing AD using a moisturizer. And lastly, the intervention in our previous RCT included not only the application of moisturizer but also a reduced frequency of bathing [8]. Bathing affects removal of materials, including allergens, from the skin. Thus, reducing the frequency of bathing might also affect allergen removal, leading to percutaneous sensitization due to prolonged exposure to allergens. However, moisturizer intervention improved the value of TEWL at 3 months of age. The current study showed that TEWL at 3 months of age was related to a subsequent food allergy until 2 years of age. Therefore, moisturizer intervention might be able to prevent a food allergy. Future research is needed to elucidate the efficacy of moisturizer or frequency of bathing for preventing allergic diseases.

Our findings confirm that a family history of AD affects the development of childhood allergic diseases. In the present study, 25–30% of children with a family history of AD developed AD or food allergies. This result is similar to a previous study that reported that 37.9% of children whose father or mother had AD developed AD before 4 years of age [12]. However, even in cases in which both parents do not have AD, approximately 8% of children develop allergic diseases (Table 1). This proportion is not negligible, but many previous studies have included only high-risk children in their study populations. Thus, researchers should also consider prevention methods among low-risk children without a family history of AD.

This study had some limitations. First, we assessed allergic diseases using a self-reported questionnaire. Thus, the definitions of food allergies and AD/eczema were not standardized. However, to avoid parents’ subjective assessments, we asked the following question: “When was AD diagnosed?” In addition, the diagnosis was not necessarily obtained from an allergy specialist, which was also a limitation of this study. Secondly, we asked parents to self-report their child’s food allergy, and we did not know whether these reported allergies had been diagnosed by certified allergists. Therefore, we have possibly overestimated food allergies. In addition, we considered multiple allergens a “food allergy” due to the small sample size; these allergens included egg, cow milk, soy, and wheat. We did not know when specific allergenic foods, like egg, had been introduced. Future research should investigate the relationship between skin problems in early infancy and food allergies in early childhood with a larger sample size and analysis of specific allergens. Lastly, although we categorized the duration of skin problems into 3 categories, there was no available evidence for determining these cut off values. We defined “short duration” as having skin problems 3–10 days in the sensitivity analysis. The results of the sensitivity analysis were similar to “short duration defined 3–7 days. Despite this limitation, we showed the novel result that “short-term skin problems” within 1 week were related to subsequent allergic diseases.

## Conclusions

This study demonstrated a relationship between the development of allergic diseases until age of 2 years and short-term skin problems during the first 0–3 months of life among infants in the general population. Future studies are needed to investigate the effective skincare methods for the prevention or prompt treatment of skin problems in early infanthood.

## Supplementary information

**Additional file 1: Table S1.** Details of food allergens. **Table S2.** Relationship between food allergy or AD/eczema until 2 years old and applying moisturizer in the first 3 months of life. **Table S3.** Risk factors significantly associated with food allergies including TEWL at 3 months of age. **Table S4.** Risk factors significantly associated with food allergies including TEWL at 1 month of age.

## Data Availability

The datasets used and analyzed during the current study are available from the corresponding author on reasonable request.

## Abbreviations

AD: atopic dermatitis
RCT: randomized control trial
TEWL: transepidermal water loss
