# Supplementary material for: Short-term skin problems in infants aged 0–3 months affect food allergies or atopic dermatitis until 2 years of age, among infants of the general population
Source: Allergy Asthma Clin Immunol. 2019 Nov 26;15:74. doi: 10.1186/s13223-019-0385-7 (PMC6880577; doi:10.1186/s13223-019-0385-7)
Supplement: Supplementary file 1 — Additional file 1: Table S1. Details of food allergens. Table S2. Relationship between food allergy or AD/eczema until 2 years old and applying moisturizer in the first 3 months of life. Table S3. Risk factors significantly associated with food allergies including TEWL at 3 months of age. Table S4. Risk factors significantly associated with food allergies including TEWL at 1 month of age. [file 13223_2019_385_MOESM1_ESM.pdf]

Table S1: Details of food allergens

Allergen	n
Egg only	11
Cow milk only	2
Peanuts only	1
Egg &cow milk	3
Egg & wheat	1
Egg & cow milk & wheat	1
Egg & cow milk & soy	1
Egg & cow milk & soy & arabesque greenling	1
Egg & cow milk & banana & almond & kiwi	1
Total	22

Table S2: Relationship between food allergy or AD/eczema until 2 years of age and applying moisturizer in the first 3 months of life

	Total	Moisturizer group†	Control group	*p*-value‡
	n=155	n=91	n=64	
Food allergy	22 (14.2)	11 (12.1)	11 (17.2)	0.370
AD/eczema	28 (18.1)	15 (16.5)	13 (20.3)	0.542

Data are presented as n (%)

AD: Atopic dermatitis

†: Moisturizer group applied moisturizer for their infants for an average of 0.7 times per day in the first 3 months of life

‡: Calculated using the Chi-square test

Table S3. Risk factors significantly associated with food allergies including TEWL at 3 months of age (n=153)

Risk factors	COR	95% CI	*p–value*	AOR[‡]	95% CI	*p–value*
Family history of AD	4.12	1.62–10.47	0.003	4.54	1.59–12.99	0.005
TEWL ($g/m^2/h$) at 3 months of age: Face	1.11	1.05–1.17	< 0.001	1.11	1.05–1.17	0.001
Solid food started after 6 months of age	2.96	1.03–8.51	0.044	2.62	0.81–8.43	0.107

COR=Crude Odds Ratio, 95% CI=95% Confidence Interval

[†]: Assessed using logistic regression analysis (food allergy, n=22; no food allergy, n=131); n=2 were missing information about when the infant began to eat complementary food

[‡]: AOR=Adjusted Odds Ratio (adjusted for 3 variables in this table)

Table S4. Risk factors significantly associated with food allergies including TEWL at 1 month of age (n=153)

Risk factors	COR	95% CI	*p–value*	AOR[‡]	95% CI	*p–value*
Family history of AD	4.12	1.62–10.47	0.003	4.46	1.66–12.00	0.003
TEWL ($g/m^2/h$) at 1 month of age: Face	1.07	1.02–1.13	0.008	1.07	1.01–1.13	0.013
Solid food started after 6 months of age	2.96	1.03–8.51	0.044	3.16	1.02–9.80	0.047

COR=Crude Odds Ratio, 95% CI=95% Confidence Interval

[†]: Assessed using logistic regression analysis (food allergy, n=22; no food allergy, n=131); n=2 were missing information about when the infant began to eat complementary food

[‡]: AOR=Adjusted Odds Ratio (adjusted for 3 variables in this table)
